# A low-temperature operated *in situ* synthesis of TiC-modified carbon nanotubes with enhanced thermal stability and electrochemical properties†

**DOI:** 10.1039/d2na00059h

**Published:** 2022-05-10

**Authors:** Huanhuan Du, Yurong Wang, Dongyang Xiao, Yili Zhang, Fangjing Hu, Leimeng Sun

**Affiliations:** School of Optical and Electronic Information, Huazhong University of Science and Technology Wuhan 430074 China sunleimeng@hust.edu.cn; MOE Key Laboratory of Fundamental Physical Quantities Measurement, Hubei Key Laboratory of Gravitation and Quantum Physics, PGMF, School of Physics, Huazhong University of Science and Technology Wuhan 430074 China fangjing_hu@hust.edu.cn

## Abstract

Carbon nanotubes (CNTs) with superior thermal and electrochemical properties are desirable for a large variety of applications. Herein, an *in situ* synthesis carried out at 1050 °C is proposed for the realization of titanium carbide (TiC) modified CNTs (TiC@CNTs) *via* a carbothermal treatment of the TiO_2_-coated CNTs deposited by a TALD technology, preserving the structural morphologies of CNT samples. Crystalline and amorphous TiC layers/nanoparticles are observed around the walls of CNTs, serving as a thermal insulation layer to enhance the thermal stability of CNTs. The TiC@CNT sample exhibits a minimal mass loss of 3.1%, which is 20.9% and 82.3% for the TiO_2_@CNT and pristine-CNT samples, respectively. In addition, the TiC@CNT electrode shows good energy storage performances, with a specific capacitance of 2.83 mF cm^−2^ at 20 μA cm^−2^, which is about 3.5 times higher than that of the pristine-CNT electrode, showing the potential of TiC@CNTs as next-generation electrode materials.

## Abstract 2

A low-temperature operated *in situ* synthesis of TiC-modified CNTs is demonstrated to improve the thermal stability and electrochemical properties of CNTs.

## Graphical abstract

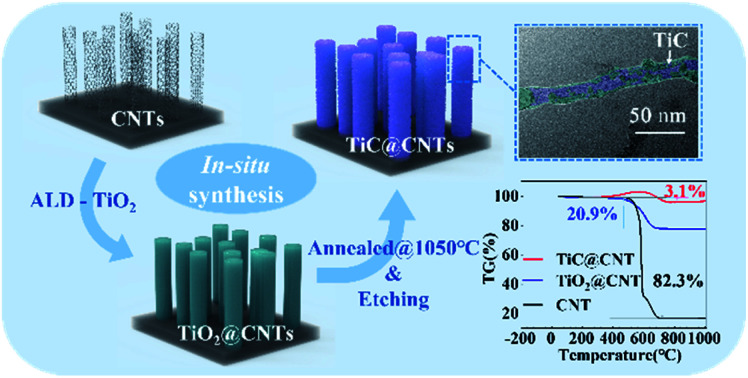

## 1. Introduction

Carbon nanotubes (CNTs) with ever improving properties have drawn extensive interest for various applications.^1^ However, the thermal and electrochemical properties of CNTs are still far below expectation due to the structural defects, metal impurities and carbon phases of CNTs originating during the fabrication process.^2,3^ In the past few decades, modification of CNTs using polymer/CNTs,^4,5^ metals/CNTs,^6^ nitrogen doped CNTs,^7^*etc.* has been demonstrated as a basic strategy to improve the thermal and electrochemical properties. However, owing to the poor conductivity of doped materials or the damaged structures caused during the synthesis process, the thermal oxidation resistance and electrochemical properties have not been significantly enhanced. Recently, two-dimensional (2D) MXene materials have shown excellent electrical, thermal and mechanical properties, and have been considered promising candidates for the functionalization of CNTs.^8,9^ In particular, titanium carbide (TiC)-based materials have been employed for numerous applications due to their high melting point, good oxidation resistance, superior mechanical properties and high electrical conductivity.^10,11^ Indeed, TiC-modified CNTs show a better thermal stability than pristine-CNTs, enabling TiC to be used as a structural reinforcement phase in composite materials.^12^

Previous studies have shown that the thermal and electrochemical performances of TiC-modified CNT (TiC@CNT) nanomaterials are associated with the size, morphology and structure of TiC@CNTs, and are crucial for applications in mechanical and electronic devices.^13,14^ So far, 1D TiC@CNT nanomaterials, such as nanowires, nanofibres, and nanotubes, have been demonstrated by methods including CNT-confined reaction,^15,16^ chemical vapor deposition,^17^ reactive spark plasma sintering,^18^ and catalysis-assisted carbothermal reduction.^19–21^ Most of the reported approaches, however, are only suitable for the preparation of powder samples of TiC@CNT nanomaterials. For example, TiC@CNT nanowires were synthesized from ball-milled powder precursors using a carbothermal reduction technique.^21^ Therefore, the nanostructures of CNT samples will be destroyed by such mechanical mixing approaches, which is not suitable for the preparation of TiC@CNTs with a specific morphology (*e.g.*, networks, arrayed structures). To overcome this limitation, *in situ* synthesis methods have been investigated for the fabrication of 1D TiC@CNT nanostructures.^22,23^ For example, the atomic layer deposition (ALD) method was applied to uniformly coat titanium oxide (TiO_2_) onto the surface of carbon nanofibres to obtain tubular TiC@CNT fibres,^22^ while chemical solution deposition was used to assist Ti precursor solutions to penetrate into the spun CNT fibres for *in situ* growth of TiC/CNT hybrid fibres.^23^ These methods can effectively avoid the damage of structural morphologies of CNT samples by harsh mechanical mixing processes. However, during the growth of TiC, a high synthesis temperature up to 1200 °C, or a demanding solution method, is normally needed, which is inconvenient and may result in the agglomeration and entanglement of CNTs. Therefore, a convenient and low-temperature operated *in situ* growth method for the preparation of TiC@CNT nanostructures with preserved structural morphology is particularly required.

In this paper, a convenient and low-temperature-operated *in situ* carbothermal reduction approach for the modification of CNT nanostructures using TiC is proposed and experimentally demonstrated to enhance the thermal stability and electrochemical properties of pristine-CNTs. The *in situ* growth approach avoids structural damage to CNT samples during the harsh ball milling process, preserving the morphology of CNT nanostructures. The thickness and morphology of the TiC layer can be easily controlled by varying the deposition thickness of TiO_2_ and the carbonization time. Compared with previously reported powder-based fabrication methods,^24,25^ TiC@CNT nanostructures can be fabricated at a relatively lower temperature of 1050 °C using the ALD-assisted method. The thermal stability evaluated by thermogravimetric (TG) analysis shows a significant improvement, from an 82.3% mass loss for pristine-CNTs, to a much lower value of 3.1% for TiC@CNTs. In addition, the assembled TiC@CNT three-electrode (*vs.* Ag/AgCl) structure exhibits typical behaviours of electric double layer capacitors (EDLCs), and a 3.5 times higher specific capacitance compared with the pristine-CNT electrode. This work provides an alternative approach for the modifications of pristine-CNTs to improve the thermal and electrochemical properties of CNTs, drawing attention to metal carbide-modified CNTs for thermal and electrochemical applications.

## 2. Experimental

### 2.1. Preparation of TiC@CNT samples

A quartz substrate was first cleaned by a conventional organic cleaning process. Subsequently, a 150 nm titanium nitride (TiN) layer and a 20 nm nickel (Ni) layer, acting as the buffer layer and catalyst, respectively, were sequentially deposited on the substrate with a magnetron sputtering system (self-assembly) and an electron beam evaporation system (EB, FU-12PEB). Next, CNT networks were synthesized at 700 °C using ethanol as the carbon source, and a mixed gas flow of 200 sccm argon (Ar) and 50 sccm hydrogen (H_2_) as the carrier gas in a thermal chemical vapor deposition (TCVD) system (OTF-1200X).

For the pristine-CNT electrodes, instead of using a TiN layer, a carbon film was prepared as the conductive layer and buffer layer. An AZ5214 photoresist was spin-coated on a Si substrate after organic and oxygen cleaning. Next, the photoresist was pre-baked at 300 °C for 1 h and annealed at 1050 °C for 4 h in the TCVD furnace with a mixed gas flow of H_2_ and Ar to obtain the carbon film. Finally, the CNT networks for the pristine-CNT electrodes were grown on the carbon film with a 20 nm Ni catalyst layer *via* the TCVD system.

The TiO_2_@CNT samples were then prepared by coating a TiO_2_ layer onto the pristine-CNT networks, using a thermal atomic layer deposition (TALD) system (TALD-611RL) at 200 °C under 0.15 Torr chamber pressure, with tetrakis(dimethylamino)titanium (TDMAT) as a titanium precursor and water (H_2_O) as an oxygen precursor. The TDMAT precursor was heated to 75 °C, while the H_2_O remained at room temperature. In this process, purge nitrogen (N_2_, 99.9999%) was used as the carrier gas. A unit growth cycle includes a TDMAT pulse (0.1 s), N_2_ purge (40 s), H_2_O pulse (0.04 s), and N_2_ purge (40 s). The thickness of the TiO_2_ layer can be controlled by the number of cycles with a growth rate of ∼0.5 Å per cycle. Samples deposited with 100 cycles (5 nm) and 400 cycles (20 nm), which are defined as the TiO_2_@CNT-5nm and TiO_2_@CNT-20nm samples, respectively, were used for later material characterization, as well as thermal stability and electrochemical property measurements.

Finally, the TiO_2_@CNT samples were annealed using a carbothermal reduction process within the TCVD system to obtain the TiC/TiO@CNT samples, which are denoted as TiC/TiO@CNT-5nm and TiC/TiO@CNT-20nm, respectively, according to the thickness of the coated TiO_2_ layer. The TiO_2_@CNT samples obtained in previous steps were placed into a sealed tube furnace that was emptied with argon for 5 min to remove the air and moisture. Subsequently, the temperature of the furnace was increased to 1050 °C at a rate of 20 °C min^−1^ and maintained for 1 h, 2 h, and 4 h in a H_2_ and Ar (1 : 4 v/v) atmosphere, respectively. After the annealing process, the TiC/TiO@CNT samples in the furnace were cooled down to room temperature, etched with diluted hydrofluoric acid (HF : H_2_O = 1 : 5 v/v), rinsed with deionized water and dried with nitrogen, to obtain the TiC@CNT samples for thermal stability and electrochemical property measurements.

### 2.2. Materials characterization

The microstructural characterization of the TiC/TiO@CNT sample was carried out using a field-emission transmission electron microscope (FETEM, Tecnai G2 F30). The sample was dropped on a micro-grid copper grid after being sonicated in ethanol solution for 10 min. The morphologies of the pristine-CNT, TiO_2_@CNT, TiC/TiO@CNT and TiC@CNT samples were characterized using a scanning electron microscope (SEM, Sigma) operating at 10 kV. Raman spectroscopy with a 532 nm argon laser (Raman, inVia Reflex) was further used to evaluate the structural integrity of CNTs before and after modification. The phase information of the TiC/TiO@CNT and TiC@CNT samples annealed for 4 h was obtained by X-ray diffraction (XRD, X'Pert Pro MPD). The surface elemental compositions of the TiC/TiO@CNT and TiC@CNT samples with 4 h annealing time were measured by an X-ray photoelectron spectrometer (XPS, AXIS-ULTRA DLD-600W).

### 2.3. Thermal stability and electrochemical measurements

To conduct a fair comparison of the contribution of TiC and TiO_2_ to the thermal stability and electrochemical properties of CNTs, and confirm that the enhanced properties come from the TiC, the TiC@CNT-20nm samples after HF solution etching were used for thermal stability and electrochemical measurements. The thermal stabilities of the pristine-CNT, TiO_2_@CNT-20nm and TiC@CNT-20nm samples were evaluated by thermogravimetry and differential scanning calorimetry (TG-DSC, STA 449 F3) with a heating rate of 10 °C min^−1^ in a synthetic air atmosphere.

The electrochemical measurements of the pristine-CNT, TiO_2_@CNT-20nm, TiC/TiO@CNT-20nm and TiC@CNT-20nm electrodes were carried out in a three-electrode electrochemical cell, which was assembled in 1 mol L^−1^ Na_2_SO_4_ aqueous solution. A platinum wire and Ag/AgCl electrode were used as the counter and reference electrodes, respectively. The electrochemical properties were investigated using a CHI 660E electrochemical workstation by measuring the cyclic voltammetry (CV), electrochemical impedance spectroscopy (EIS) and galvanostatic charge–discharge (GCD). The EIS measurements were performed within the 0.01 Hz to 100 kHz frequency range, with an amplitude of 5 mV.

## 3. Results and discussion

**Fig. 1:**
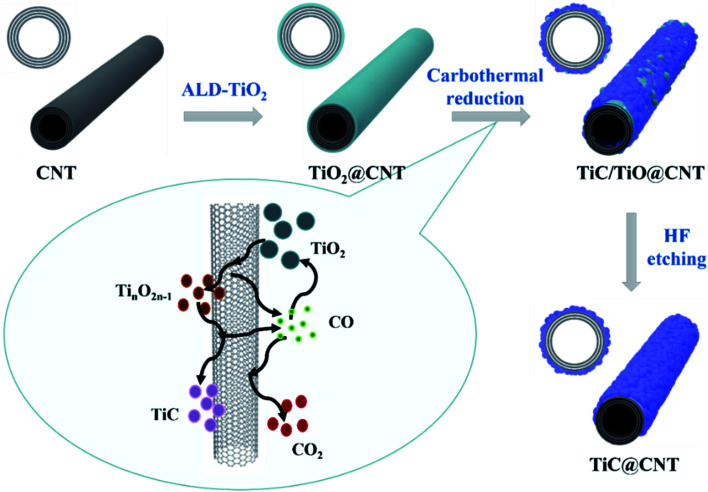
The formation mechanism of TiC@CNT composite materials.

The growth mechanism of the TiC@CNT material is illustrated in Fig. 1. The pristine-CNTs were first grown on a quartz substrate by TCVD. Next, TALD was used to deposit a uniform TiO_2_ layer with a controlled thickness onto the CNTs. Following that, the TiO_2_@CNT samples were annealed at 1050 °C for different times in a H_2_/Ar atmosphere. Finally, the TiC/TiO@CNT sample was etched with HF solution to obtain the TiC@CNT sample. The overall carbothermal reaction process is represented as follows:^26,27^

1 TiO_2_(s) + 3C(s) = TiC(s) + 2CO(g)

The reaction steps presented in Fig. 1 can be denoted by the following reactions:

2 *n*TiO_2_(s) + C(s) = Ti_*n*_O_2*n*−1_(s) + CO(g)

3 *a*Ti_*n*_O_2*n*−1_(s) + *b*C(s) = *c*TiC_*x*_O_*y*_(s) + *d*CO(g)

4 *a*TiC_*x*_O_*y*_(s) + *b*C(s) = *c*TiC(s) + *d*CO(g)

5 CO(g) + *n*TiO_2_(s) = Ti_*n*_O_2*n*−1_(s) + CO_2_(g)

6 CO_2_(g) + C(s) = 2CO(g)

In the initial stage, TiO_2_ reacts with amorphous carbon/CNTs and is reduced to Ti_*n*_O_2*n*−1_ as described by eqn (2). As the reaction continues from eqn (3) to eqn (4), the carbon sources gradually react with Ti_*n*_O_2*n*−1_ to form TiC_*x*_O_*y*_ and can be further reduced to TiC. These reaction steps generate the gaseous by-product CO, which not only plays an important role in the transition processes from TiO_2_ to TiC, as shown in eqn (5) and (6), but also induces the formation of TiC nanoparticles.^28,29^

**Fig. 2:**
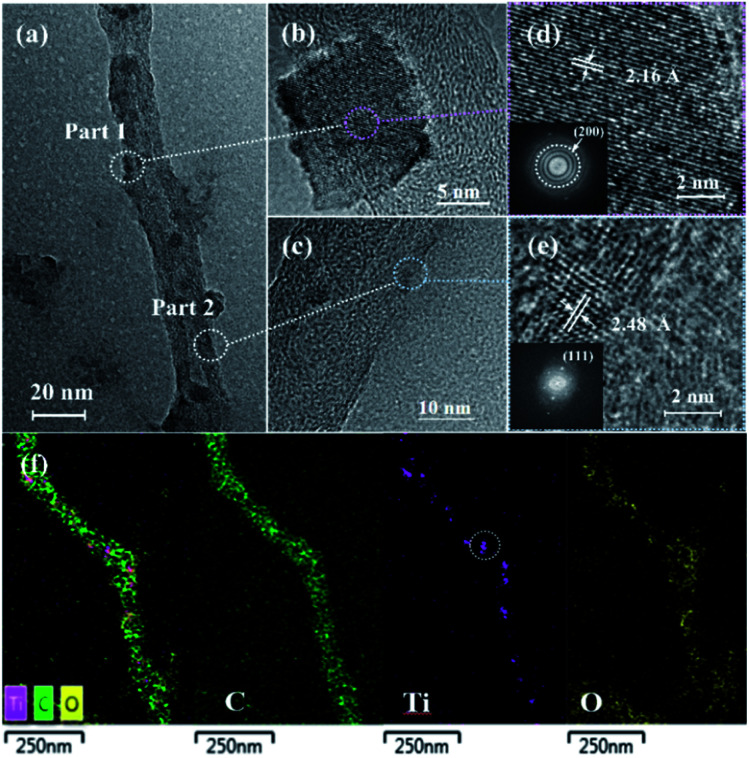
(a) The TEM image and (b and c) the zoomed-in TEM images of the TiC/TiO@CNT-5nm sample annealed at 1050 °C for 4 h. (d and e) The HRTEM images of the two marked areas with the inserted SAED patterns. (f) The element mapping of the TiC/TiO@CNT-5nm sample.

The TEM images of the TiC/TiO@CNT-5nm sample annealed at 1050 °C for 4 h are presented in Fig. 2a to c. The corresponding spacing between two adjacent lattice fringes of the two marked areas is 2.16 Å and 2.48 Å, respectively, as shown in the high-resolution TEM (HRTEM) images of Fig. 2d and e. The two measured lattice fringes are consistent with the (200) and (111) planes of cubic TiC,^30^ respectively, revealing the successful synthesis of TiC nanomaterials. Furthermore, TiC crystalline structures can be identified by selected area electron diffraction (SAED) patterns (provided as insets in Fig. 2d and e). In addition, amorphous areas of TiC are also observed around these nanoparticles on the walls of CNTs, which indicates that amorphous and crystalline TiC were formed on the surface of CNTs in both layered and granular morphologies. The same results are shown by the TEM images of the TiC/TiO@CNT-20nm sample, as shown in Fig. S1,† which further confirms that the TiC/Ti_*n*_O_2*n*−1_-modified CNTs are successfully synthesized by the *in situ* method at 1050 °C. Compared with powder samples, a lower growth temperature is required due to the larger contact area between TiO_2_ and carbon sources, and smaller TiO_2_ grains obtained by the TALD technology. Moreover, the CNTs with amorphous carbon grown by TCVD provide more active carbon atoms for the growth of TiC than the pure CNTs,^31^ which is beneficial to the carbothermal reaction. The elemental mapping of the TiC/TiO@CNT-5nm sample demonstrates a tubular morphology of nanoparticle-coated CNTs, as shown in Fig. 2f. From the element distribution, TiC nanoparticles (the circled area in Fig. 2f) and titanium oxides are observed.

**Fig. 3:**
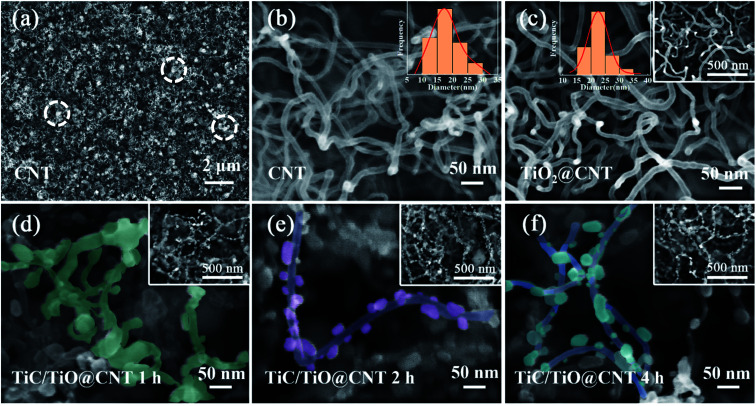
(a and b) SEM images of the pristine-CNT network with amorphous carbon. (c) SEM image of the TiO_2_@CNT-5nm sample. (d, e and f) SEM images of the TiC/TiO@CNT-5nm samples with 1 h, 2 h, and 4 h annealing time, respectively.

The morphologies of CNTs before and after modification were characterized by SEM. As shown in Fig. 3a and b, the pristine-CNT network with amorphous carbon (the white circled areas) is observed, and the diameter of CNTs is within 10–30 nm. After the ALD process, the TiO_2_@CNT-5nm sample shows an increased diameter (15–35 nm), as well as a smooth surface morphology, as exhibited in Fig. 3c, confirming the uniform decoration of the ∼5 nm TiO_2_ layer on CNTs. The SEM images of the TiC/TiO@CNT-5nm samples annealed for 1 h, 2 h and 4 h, respectively, are demonstrated in Fig. 3d–f. As the annealing time increases, the TiO_2_-coated CNTs gradually transform into TiC/Ti_*n*_O_2*n*−1_ nanoparticle-modified CNTs, and the agglomeration of nanoparticles on CNTs becomes more significant. These phenomena are also observed in the TiC/TiO@CNT-20nm samples (Note S2 and Fig. S2†). The formation of these nanoparticles is due to two reasons. First, the CO gas released by the reaction of TiO_2_/Ti_*n*_O_2*n*−1_ and carbon sources increases the roughness of the CNT walls, and results in porous structures of the TiC/TiO@CNT nanomaterials.^27^ The other reason is attributed to the high heating rate,^32,33^ which results in a lower melting temperature of TiO_2_/TiC when compared with the reaction temperature, causing the formation of nanoparticles. In addition, the SEM images shown in Fig. S3† demonstrate that the surface roughness of the TiC@CNT-20nm sample increases after HF etching, indicating that the larger active surface area of TiC is obtained after the etching of TiO_2_, which is beneficial to the electron/ion transportation of the interface of the electrode/electrolyte.^34^ The structural morphologies of CNT networks are not changed *via* the *in situ* synthesis method, so it can also be used for the modification of arrayed CNTs.

**Fig. 4:**
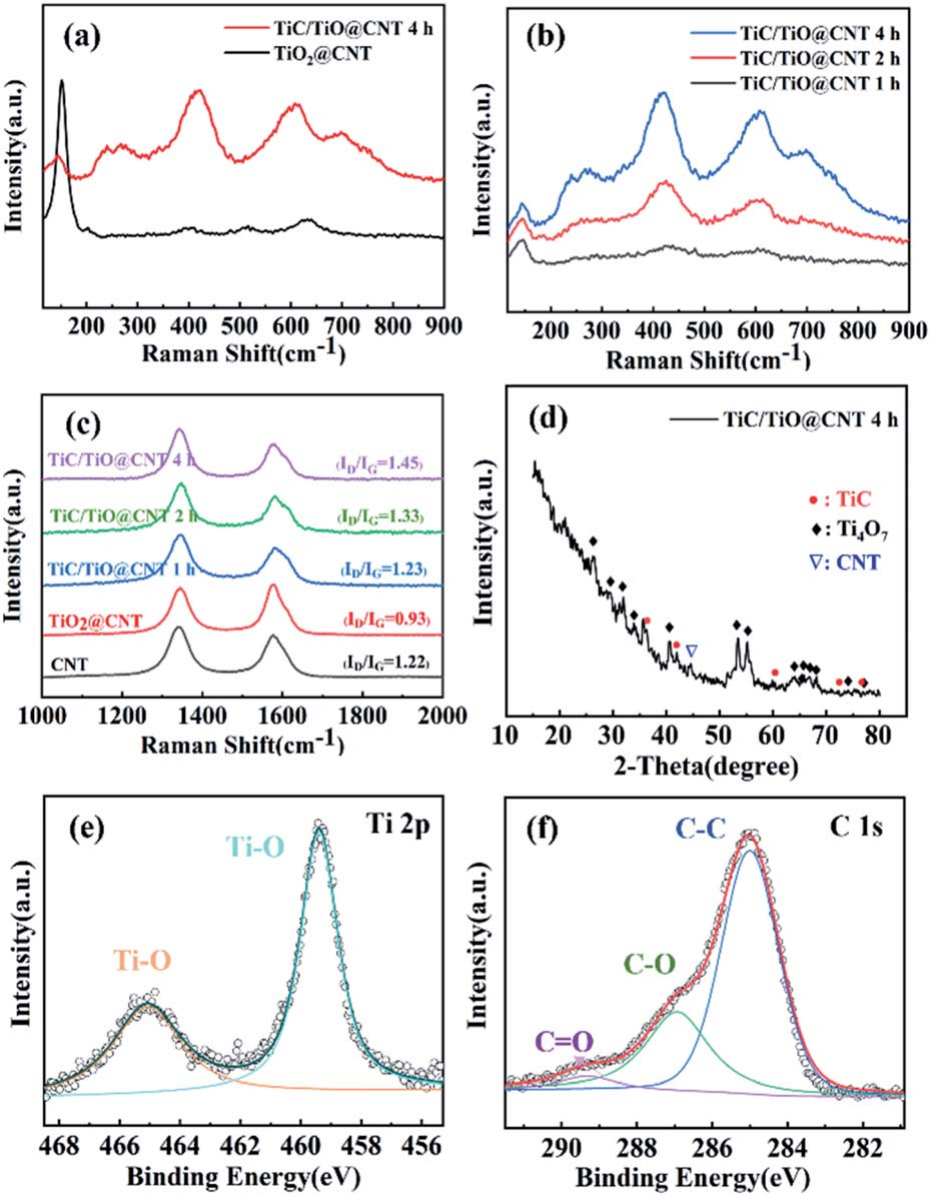
Raman spectra of (a) the TiO_2_@CNT-5nm samples unannealed and annealed for 4 h, (b) the TiC/TiO@CNT-5nm samples annealed for 1 h, 2 h and 4 h, and (c) the untreated and treated CNT samples. (d) X-ray diffraction spectrum of the TiC/TiO@CNT-5nm sample. XPS spectra of (e) the Ti 2p peak and (f) C 1s peak of the TiC/TiO@CNT-5nm sample.

Raman spectroscopy was performed to further validate the formation of the TiC phase and the structural integrity of CNTs. Fig. 4a shows that the TiO_2_ Raman peaks are located at 147 cm^−1^, 397 cm^−1^, 511 cm^−1^, and 629 cm^−1^, which correspond to the anatase phase of TiO_2_.^35^ After annealing for 4 h for the TiO_2_@CNT sample, the TiO_2_ peaks partially disappear and TiC peaks located at 260 cm^−1^, 415 cm^−1^, and 609 cm^−1^ are observed.^36^ The shift of Raman peaks is consistent with the results of the TiC/TiO@CNT-20nm sample (Fig. S4a†). In addition, as the annealing time increases, the Raman intensity of the TiC peaks enhances under the same test conditions (Fig. 4b and S4b†), revealing that more TiC materials are synthesized. It is believed that the remaining titanium oxides can continuously convert into TiC if the annealing time further increases. The structural integrity of the untreated and treated CNTs was evaluated by the *I*_D_/*I*_G_ intensity ratio associated with structural defects.^37^ As shown in Fig. 4c, the *I*_D_/*I*_G_ value of the pristine-CNT sample is 0.9 and it increases to >1.3 after annealing, indicating that the walls of CNTs have reacted and are damaged during carbothermal reduction. Furthermore, the *I*_D_/*I*_G_ values of TiC/TiO@CNT-5nm samples annealed for 1 h, 2 h, and 4 h are around 1.31, 1.35, and 1.45, respectively. In other words, with the increase of reaction time, the defects of the TiC/TiO@CNT-5nm sample increase, due to the continuous reaction between the walls of CNTs and TiO_2_ to form TiC.

The X-ray diffraction pattern of the TiC/TiO@CNT-5nm sample with a 4 h annealing time is illustrated in Fig. 4d. The diffraction peaks of a lower oxide of titanium (Ti_4_O_7_) (JCPDS 50-0787) are observed, which can be considered as an intermediate phase for the transformation of TiO_2_ into TiC.^38^ The nanostructured TiC peaks at 35.67°, 41.6° and 76.5° can be indexed to the (111), (200) and (222) reflections of cubic TiC (JCPDS 65-0242).^39^ These diffraction peaks are also observed in the TiC/TiO@CNT-20nm sample, as shown in Fig. S5a.† In comparison, the titanium oxidation peaks of the TiC@CNT-20nm sample are reduced in intensity or have disappeared. However, the relative intensities of TiC peaks are low and almost unchanged, which is attributed to the formation of amorphous TiC caused by the crystallization of amorphous carbon induced by the heat treatment. In general, the reaction between TiO_2_ and crystalline carbon occurs at a high temperature above 1278 °C, because the crystalline carbon has a lower reactivity than amorphous carbon.^31^

XPS spectra were used to characterize the surface elemental compositions of the TiC/TiO@CNT-5nm sample. The Ti 2p core level spectrum in Fig. 4e can be resolved into two peaks centred at 465.1 eV and 459.4 eV which originate from Ti 2p_3/2_ and Ti 2p_1/2_ electrons in titanium oxide,^40,41^ respectively. For the C 1s spectrum (Fig. 4f), the predominant peak at 285.0 eV corresponds to the C–C bond, and the low intensity peaks at 286.9 eV and 289.3 eV correspond to the C–O bond and C=O bond, respectively.^42^ The C–Ti bond is, however, not observed, because TiC is partially amorphous and is not distributed on the superficial layer of the sample. Similar results are also found in the TiC/TiO@CNT-20nm sample, as shown in Fig. S5b and c.† In addition, the F 1s core level spectrum of the TiC@CNT-20nm sample at 685.0 eV is observed after HF etching, indicating that F^−^ ions are physically adsorbed on the surface of the film, rather than F being doped into TiO_2_, which should have an XPS peak at approximately 688 eV.^43,44^

**Fig. 5:**
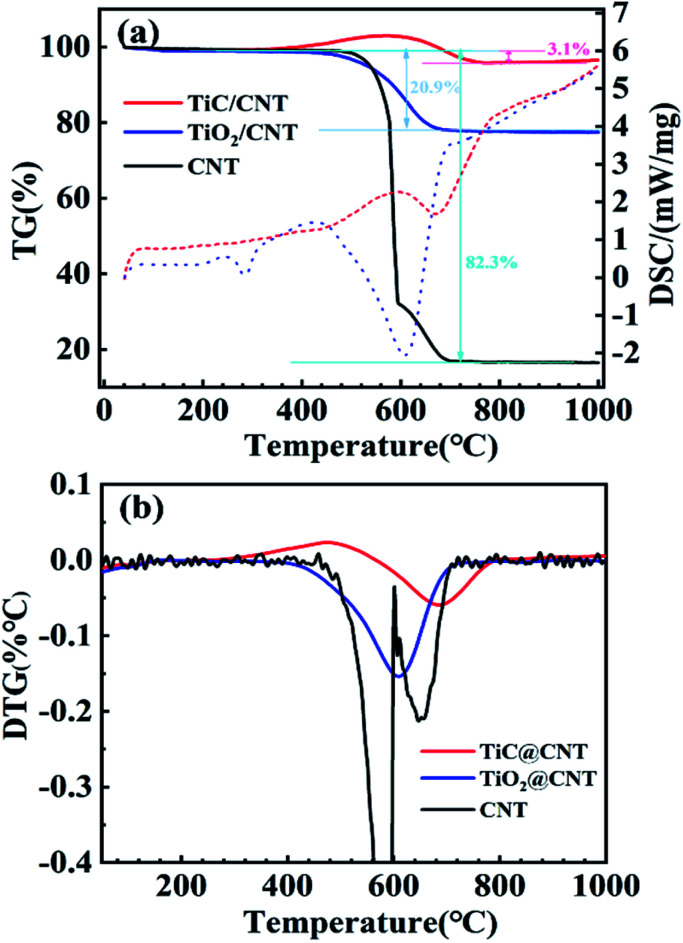
(a) TG-DSC analysis (solid and dotted lines represent TG and DSC curves, respectively) and (b) DTG curves of the untreated and treated samples.

The thermal stabilities of the pristine-CNT, TiO_2_@CNT and TiC@CNT samples were investigated by thermogravimetric and differential scanning calorimetric (TG-DSC) analysis. To obtain more active material loading, the TiO_2_@CNT-20nm sample was used for the measurements. For the thermogravimetric (TG) curves shown in Fig. 5a, an increase in mass was observed for the TiC@CNT sample between around 370 °C and 600 °C, corresponding to the oxidation of the TiC layer,^45,46^ further revealing the formation of TiC. As the temperature increases to >600 °C, the TiC@CNT sample presents a mass loss, as the burning of CNTs^47^ introduces a larger mass loss than the mass gain from the oxidation of TiC. The pristine-CNT sample starts to burn at around 470 °C with a sharp slope, and shows a mass loss of 82.3%. In comparison, the TiO_2_@CNT-20nm sample shows a lower mass loss of 20.9%, due to the protection of the outer coating layer on the surface of CNTs.^48^ The TiC@CNT-20nm sample displays a minimal mass loss of only 3.1%, as well as a minimal slope among the three samples, which is consistent with those of the differential scanning calorimetry (DSC) curves depicted in Fig. 5a. The derivative thermogravimetry (DTG) curves in Fig. 5b show that the oxidation peaks of these three structures appear successively at around 580 °C, 610 °C and 690 °C, confirming the protective effect of the coating on the surface of CNTs. Therefore, the TiC layer is regarded as a thermal insulation coating to protect CNTs from oxidation and enhance the thermal stability of CNTs, which is beneficial to the applications of TiC@CNTs in a high temperature environment.

**Fig. 6:**
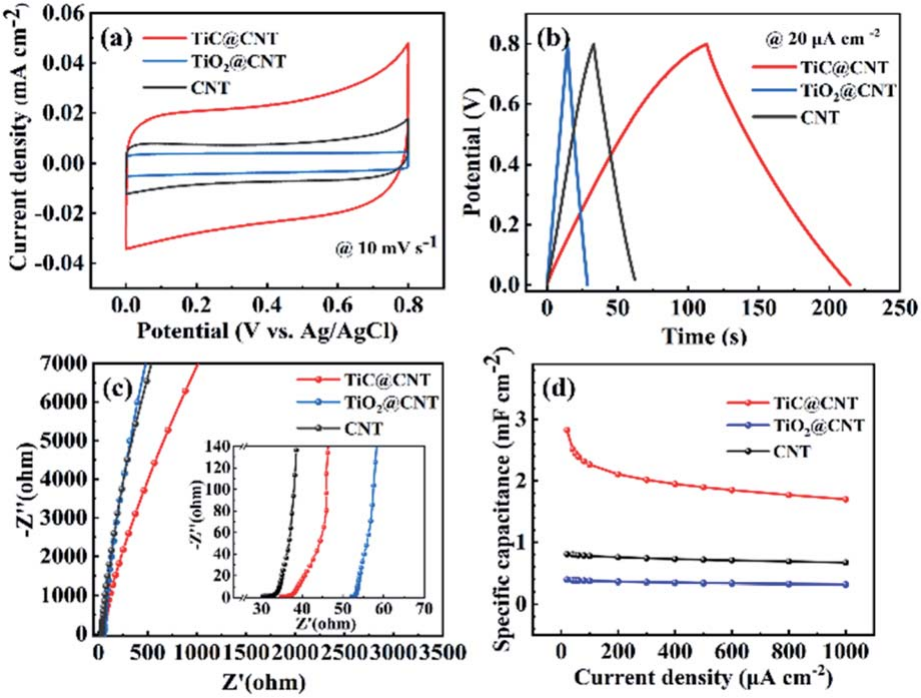
Electrochemical properties of the pristine-CNT, TiO_2_@CNT and TiC@CNT-20nm electrodes: (a) CV curves at a scan rate of 10 mV s^−1^; (b) GCD curves at a current density of 20 μA cm^−2^; (c) Nyquist plots; (d) specific capacitances.

The electrochemical properties of the pristine-CNT, TiO_2_@CNT-20nm and TiC@CNT-20nm electrodes were characterized in a three-electrode system (*vs.* Ag/AgCl) with 1 mol L^−1^ Na_2_SO_4_ aqueous solution. The CV curves (Fig. 6a) of all three electrodes exhibit quasi-rectangular shapes at a scan rate of 10 mV s^−1^, suggesting that the energy storage capability of the three electrodes arises from the typical EDLC behaviour rather than the pseudo-capacitive behaviour. Furthermore, the symmetric-shaped CV curves at scanning rates ranging from 5 to 80 mV s^−1^ further explain the EDLC behaviour of the TiC@CNT electrode (Fig. S6a†). Compared with the pristine-CNT and the TiO_2_@CNT electrodes, the TiC@CNT electrode shows a higher current density and a larger enclosed area of the CV curve at the same scan rate of 10 mV s^−1^, indicating the superior energy storage performance of the TiC@CNT electrode. This is further confirmed by the corresponding GCD curves with nearly triangular shapes as shown in Fig. 6b and S6b.† The TiC@CNT electrode has a longer discharge time, since the as-prepared TiC@CNT sample has a more porous structure and larger tube diameters than the pristine-CNT electrode, providing more active sites due to the plentiful inherent defects of the TiC amorphous phase^49,50^ and a larger surface area for electron/ion transportation.^34^ In addition, the electrochemical performances of the TiC/TiO@CNT electrode shown in Fig. S7† also confirm that the enhanced electrochemical activity of the TiC@CNT electrode is attributed to the appearance of a larger TiC active surface area after HF etching. The Nyquist plots shown in Fig. 6c demonstrate that the TiC@CNT electrode presents a gentler slope than the TiO_2_@CNT and pristine-CNT electrodes in the low frequency region, indicating a lower ion diffusivity and a higher Warburg resistance (*Z*_w_), which is caused by the increase of defects and an HF solution etching procedure.^51^

The specific capacitances of pristine-CNT, TiO_2_@CNT and TiC@CNT electrodes at different current densities are presented in Fig. 6d. The TiC@CNT electrode exhibits the highest specific capacitance of 2.83 mF cm^−2^ at 20 μA cm^−2^, which is approximately 3.5 times higher than that of the pristine-CNT electrode (0.81 mF cm^−2^), and 7.1 times higher than that of the TiO_2_@CNT electrode (0.39 mF cm^−2^), due to the larger active surface area and porous structure of the TiC@CNT sample. It is thus promising to apply metal carbide-modified CNTs as electrode materials to boost the electrochemical performances of nanostructured CNTs.

## 4. Conclusion

In this work, a low-temperature-operated *in situ* synthesis strategy for TiC-modified CNTs was proposed to enhance the thermal stability and electrochemical properties of CNTs, without damaging the structural morphologies of the CNT samples. The TiC samples were successfully synthesized at 1050 °C *via* a carbothermal reduction process of the TiO_2_@CNT samples. With an increased annealing time, more TiC/Ti_*n*_O_2*n*−1_ nanoparticles appeared on the surface of CNTs, which is mainly due to the release of by-product gas. The formation of TiC with crystalline and amorphous phases was found on the surface of CNTs in both layered and granular morphologies, serving as a thermal insulation coating to enhance the oxidation resistance of pristine-CNTs. The TiC@CNT sample showed a mass loss of only 3.1%, which was 20.9% for the TiO_2_@CNT sample and 82.3% for the pristine-CNTs, respectively. In addition, the TiC@CNT electrode exhibited a typical EDLC behaviour and superior energy storage performances to its counterparts. The specific capacitance of the TiC@CNT electrode was about 3.5 times higher than that of the pristine-CNT electrode, due to a larger surface area and more porous structure. The proposed strategy provides an alternative way to functionalize the nanostructured-CNTs, paving the way for metal carbide-modified CNTs as next-generation electrode materials.

## Conflicts of interest

There are no conflicts to declare.

## Supplementary Material

NA-004-D2NA00059H-s001
