# Supplementary material for: A low-temperature operated in situ synthesis of TiC-modified carbon nanotubes with enhanced thermal stability and electrochemical properties
Source: Nanoscale Adv. 2022 May 10;4(11):2444–51. doi: 10.1039/d2na00059h (PMC9417096; doi:10.1039/d2na00059h)
Supplement: NA-004-D2NA00059H-s001 [file NA-004-D2NA00059H-s001.pdf]

Electronic Supplementary Material (ESI) for Nanoscale Advances.
This journal is © The Royal Society of Chemistry 2022

Electronic Supplementary Information

A low-temperature operated *in-situ* synthesis of TiC-modified carbon nanotubes with enhanced thermal stability and electrochemical properties

Huanhuan Du,[a,b] Yurong Wang,[b] Dongyang Xiao,[b] Yili Zhang,[b] Fangjing Hu *[b] and Leimeng Sun *[a]

[a]School of Optical and Electronic Information, Wuhan National Laboratory for Optoelectronics, Huazhong University of Science and Technology, Wuhan 430074, China.

[b]MOE Key Laboratory of Fundamental Physical Quantities Measurement & Hubei Key Laboratory of Gravitation and Quantum Physics, PGMF and school of Physics, Huazhong University of Science and Technology, Wuhan 430074, China.

*Corresponding author: sunleimeng@hust.edu.cn (L. S.); fangjing_hu@hust.edu.cn (F. H.)

TiO_2@CNT-5nm: pristine-CNTs with 5 nm thick TiO_2 coating

TiO_2@CNT-20nm: pristine-CNTs with 20 nm thick TiO_2 coating

TiC/TiO@CNT-5nm: TiC-modified CNTs as prepared from TiO_2@CNT-5nm

TiC/TiO@CNT-20nm: TiC-modified CNTs as prepared from TiO_2@CNT-20nm

TiC@CNT-20nm: The TiC/TiO@CNT-20nm sample after HF solution etching

Note S1: TEM images of the TiC/TiO@CNT-20nm sample with a 4 h annealing time

The transmission electron microscope (TEM) images of the TiC/TiO@CNT-20nm sample with a 4 h annealing time are shown in Fig. S1. The TiC layers/nanoparticles with crystalline and amorphous phases are observed on the surface of CNTs. The lattice fringes provided in Fig. S1b and S1c confirm the existence of (111) and (200) planes of TiC in the TiC/TiO@CNT-20nm sample[1].

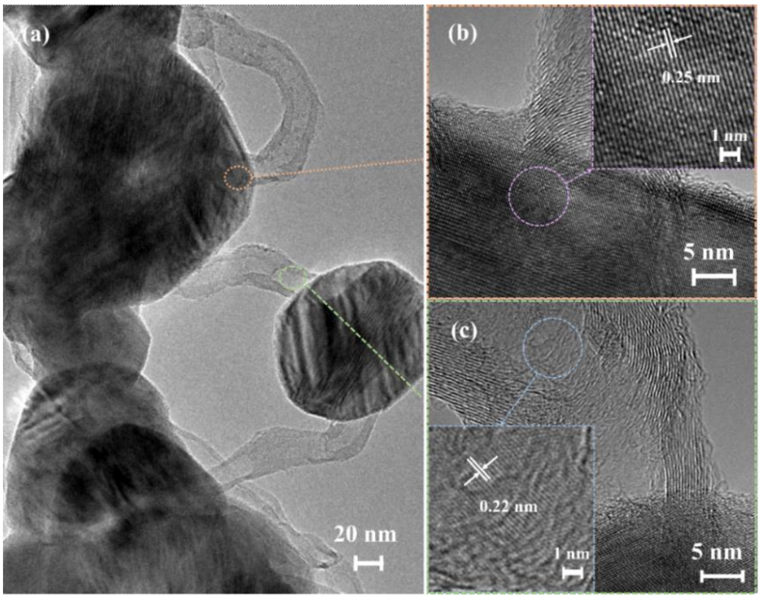

Fig. S1 (a) TEM images of TiC/TiO@CNT-20nm sample with a 4 h annealing time. (b) and (c) high-resolution TEM (HRTEM) images in different regions of TiC/TiO@CNT-20nm sample (inset: corresponding lattice fringe).

Note S2: SEM characterizations of the TiO_2@CNT-20nm and TiC/TiO@CNT-20nm samples

SEM images of the TiC/TiO@CNT-20nm samples with different annealing time confirm

that the morphologies of CNTs have changed significantly after annealing, as shown in Fig. S2. As the annealing time increases, the TiO_2 layer on the surface of CNTs gradually transforms into TiC/Ti_nO_{2n-1} nanoparticles, and the agglomerations of nanoparticles become more significant[2, 3]. Compared with the TiC/TiO@CNT-5nm sample, the TiC/TiO@CNT-20nm sample contains larger grains and more active materials.

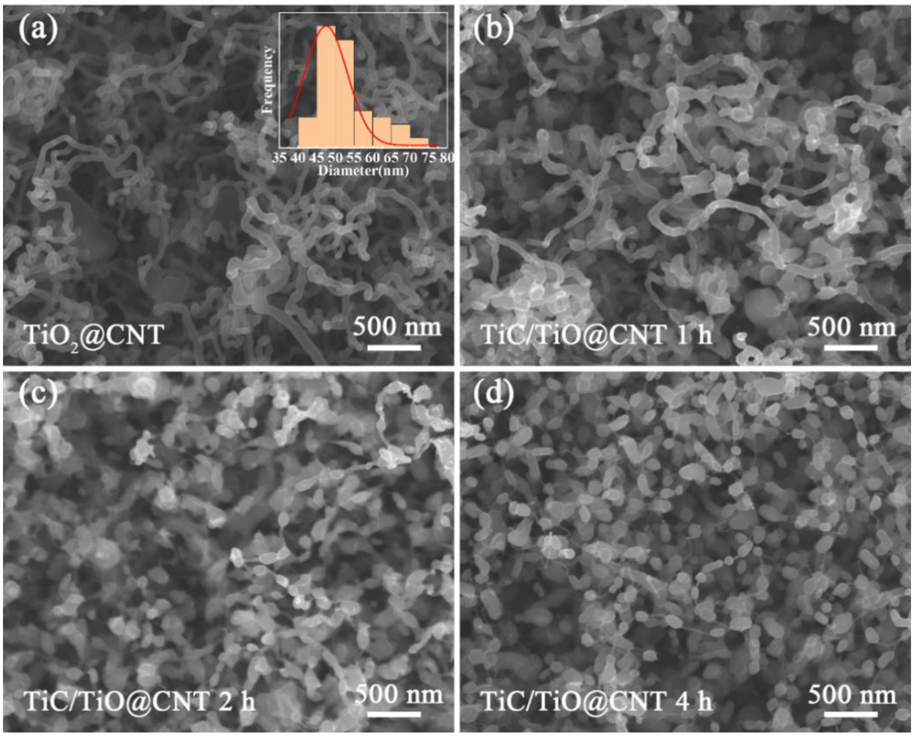

Fig. S2 SEM images of (a) TiO_2@CNT-20nm sample, and TiC/TiO@CNT-20nm samples with an annealing time of (b) 1 h, (c) 2 h, and (d) 4 h, respectively.

Note S3: SEM characterizations of the TiC/TiO@CNT-20nm and TiC@CNT-20nm samples

The morphologies for the TiC/TiO@CNT-20nm and TiC@CNT-20nm samples are shown in Fig. S3. It is found that the surface roughness of CNTs increases after HF etching, indicating a larger active surface area of TiC after the etching of TiO_2, which is beneficial to the electron/ion transportation of the electrode/electrolyte interface[4].

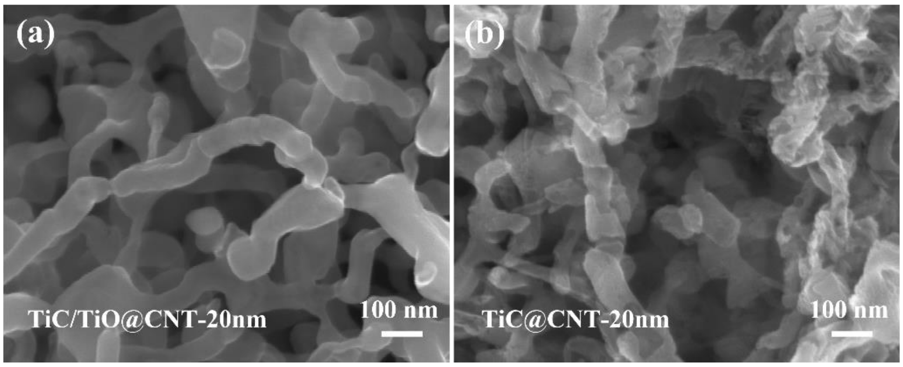

Fig. S3 SEM images of (a) TiC/TiO@CNT-20nm sample, and (b) TiC@CNT-20nm sample with a 4 h annealing time.

Note S4: Raman spectra of the unannealed and annealed TiO_2@CNT-20nm samples

Raman spectra of the TiO_2@CNT-20nm and TiC/TiO@CNT-20nm samples with an annealing time of 1 h, 2 h, and 4 h are demonstrated in Fig. S4. A shift of Raman peaks from TiO_2 to TiC is observed for the TiO_2@CNT-20nm sample after annealing[5, 6]. Moreover, as the annealing time increases, the Raman intensity of TiC peaks increases under the same test conditions, revealing the synthesis of more TiC nanomaterials.

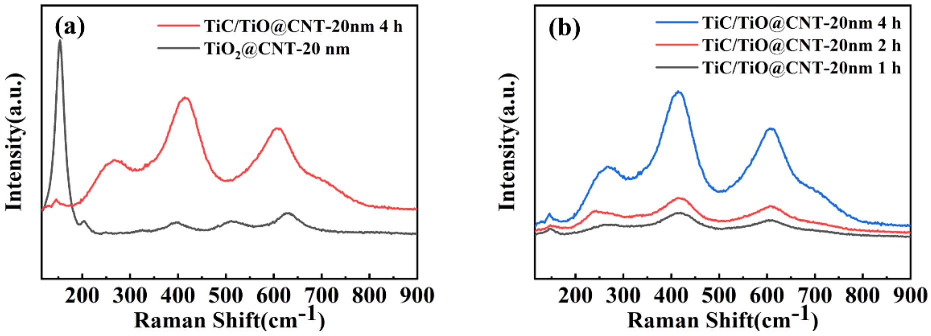

Fig. S4 Raman spectra of (a) TiO_2@CNT-20nm sample and TiC/TiO@CNT-20nm sample with a 4 h annealing time, (b) TiC/TiO@CNT-20nm samples with various annealing time.

Note S5: XRD and XPS spectra of the TiC/TiO@CNT-20nm and TiC@CNT-20nm samples

Compared with the TiC/TiO@CNT-20nm sample, the titanium oxidation diffraction peaks of the TiC@CNT-20nm sample are reduced or disappeared (Fig. S5a), revealing that the titanium oxidation has been etched by HF solution. The intensities of TiC peaks

are low and almost unchanged, due to the amorphous TiC and residual TiO_2. The Ti 2p peaks are observed in the XPS spectra of both samples, which is consistent with the results of the XRD spectra. In addition, the F 1s core level spectrum of TiC@CNT-20nm sample at 685.0 eV reveals that F^- ion is physically absorbed on the surface of the film after HF etching, rather than F doping into TiO_2[7, 8].

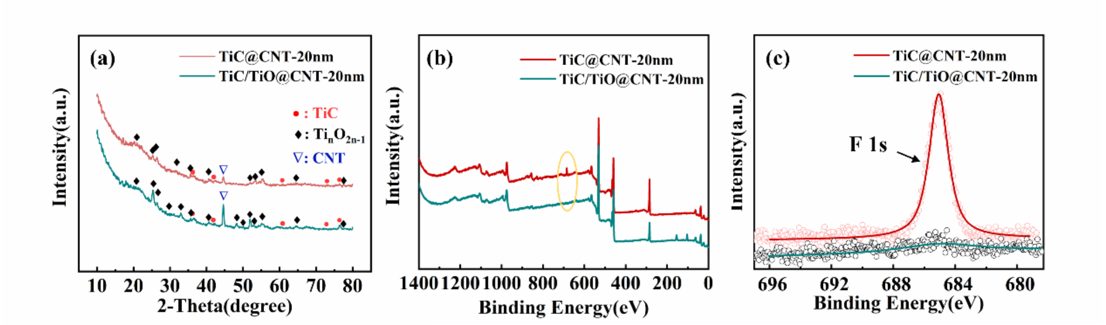

Fig. S5 (a) XRD spectra, (b) XPS spectra, and (c) high-resolution XPS spectra of F 1s region of TiC/TiO@CNT-20nm and TiC@CNT-20nm samples with a 4 h annealing time.

Note S6: Electrochemical performances of the TiC@CNT-20nm electrode with a 4 h annealing time

Fig. S6 shows symmetric cyclic voltammetry (CV) curves for the TiC@CNT-20nm electrode with a scan rate from 5 to 80 mV s^{-1}, revealing the faradic reaction at the interface of electrode/electrolyte[9]. Quasi-triangular shapes for the galvanostatic charge-discharge (GCD) curves with different current densities demonstrate superior electrochemical reversibility of the TiC@CNT-20nm electrode.

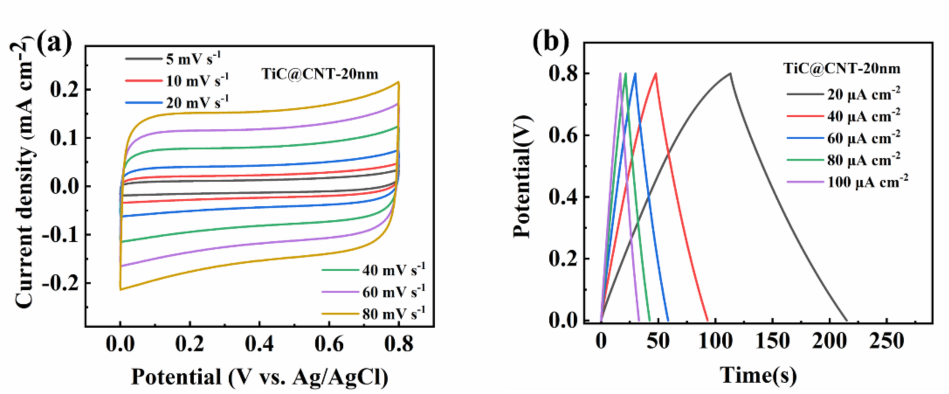

Fig. S6 Electrochemical properties of TiC@CNT-20nm electrode with a 4 h annealing time in a three-electrode system (vs. Ag/AgCl) with 1 mol L^{-1} Na_2SO_4 aqueous solution: (a) CV curves for different scan rates; (b) GCD curves with different current densities.

Note S7: Electrochemical performances of the TiC/TiO@CNT-20nm electrode with a 4 h annealing time

The symmetrical shapes of CV curves and GCD curves demonstrate that the energy storage capability of TiC/TiO@CNT-20nm electrode arises from typical EDLC behaviour. The series resistance (R_S) of TiC/TiO@CNT-20nm electrode is relatively low due to the synthesis of TiC materials.

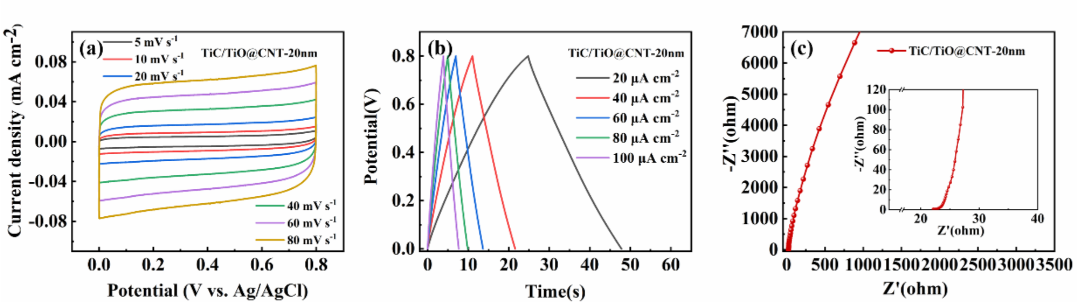

Fig. S7 Electrochemical properties of TiC/TiO@CNT-20nm electrode with a 4 h annealing time in a three-electrode system (vs. Ag/AgCl) with 1 mol L^{-1} Na_2SO_4 aqueous solution: (a) CV curves; (b) GCD curves; (c) Nyquist plot.

References

1. L. Zhang, M. S. Tse, O. K. Tan, Y. X. Wang and M. Han, *J. Mater. Chem. A*, 2013, **1**, 4497.
2. Y.-C. Woo, H.-J. Kang and D. J. Kim, *J. Eur. Ceram. Soc.*, 2007, **27**, 719-722.
3. B. Li and W. Zhang, *Ultrason. Sonochem.*, 2020, **61**, 104837.
4. M.-G. Jeong, W.-J. Kwak, H.-J. Shin, Y.-K. Sun and H.-G. Jung, *Chem. Eng. J.*, 2020, **399**, 125699.
5. L. Sun, X. Wang, Y. Wang, D. Xiao, W. Cai, Y. Jing, Y. Wang, F. Hu and Q. Zhang, *Front Chem*, 2019, **7**, 512.
6. H. An, B. Zhu, J. Li, J. Zhou, S. Wang, S. Zhang, S. Wu and W. Huang, *J. Phys. Chem. C*, 2008, **112**, 18772-18775.
7. J. C. Yu, J. Yu, W. Ho, Z. Jiang and L. Zhang, *Chem. Mater.*, 2002, **14**, 3808-3816.
8. M. Kitano, K. Iyatani, K. Tsujimaru, M. Matsuoka, M. Takeuchi, M. Ueshima, J. M. Thomas and M. Anpo, *Top. Catal.*, 2008, **49**, 24-31.
9. X. Xia, Y. Zhang, D. Chao, Q. Xiong, Z. Fan, X. Tong, J. Tu, H. Zhang and H. J. Fan, *Energy Environ. Sci.*, 2015, **8**, 1559-1568.
